# Characterization of the Pathogenic Potential of the Beach Sand Microbiome and Assessment of Quicklime as a Remediation Tool

**DOI:** 10.3390/microorganisms11082031

**Published:** 2023-08-07

**Authors:** Irene Soffritti, Maria D’Accolti, Francesca Bini, Eleonora Mazziga, Antonella Volta, Matteo Bisi, Silvia Rossi, Francesco Viroli, Marcello Balzani, Marco Petitta, Sante Mazzacane, Elisabetta Caselli

**Affiliations:** 1 Section of Microbiology, Department of Chemical, Pharmaceutical and Agricultural Sciences, and LTTA, University of Ferrara, 44121 Ferrara, Italy; irene.soffritti@unife.it (I.S.); maria.daccolti@unife.it (M.D.); francesca.bini@unife.it (F.B.); eleonora.mazziga@unife.it (E.M.); 2 CIAS Research Center, University of Ferrara, 44122 Ferrara, Italy; antonella.volta@unife.it (A.V.); matteo.bisi@unife.it (M.B.); sante.mazzacane@unife.it (S.M.); 3 Building and Construction Cluster of the Emilia Romagna Region, 40129 Bologna, Italy; silvia.rossi@build.clust-er.it; 4 TekneHub, Department of Architecture, University of Ferrara, 44121 Ferrara, Italy; francesco.viroli@unife.it (F.V.); marcello.balzani@unife.it (M.B.); 5 Department of Earth Sciences, University “La Sapienza”, 00185 Rome, Italy; marco.petitta@uniroma1.it

**Keywords:** pathogen contamination, beach sand microbiome, WGS, quicklime, decontamination

## Abstract

Beach sand may act as a reservoir for potential human pathogens, posing a public health risk. Despite this, the microbiological monitoring of sand microbiome is rarely performed to determine beach quality. In this study, the sand microbial population of a Northern Adriatic Sea beach sand was profiled by microbiological (CFU counts) and molecular methods (WGS, microarray), showing significant presence of potential human pathogens including drug-resistant strains. Consistent with these results, the potential of quicklime as a restoring method was tested in vitro and on-field. Collected data showed that adding 1–3% quicklime (*w*/*w*) to sand provided an up to −99% of bacteria, fungi, and viruses, in a dose- and time-dependent manner, till 45 days post-treatment. In conclusion, data suggest that accurate monitoring of sand microbiome may be essential, besides water, to assess beach quality and safety. Moreover, first evidences of quicklime potential for sand decontamination are provided, suggesting its usage as a possible way to restore the microbiological quality of sand in highly contaminated areas.

## 1. Introduction

Exposure to environmental pathogens is a common human health risk in beaches, due to the possibly high levels of microbial contamination detected in the beach sand. Beach sand may in fact act as a reservoir for several microbes potentially pathogenic to humans, especially in highly populated and industrialized areas, and microbiological monitoring of beach sand may be an important tool to assess the sand microbiological quality, whereas only beach quality monitoring is usually performed by assessing only water quality.

Diseases can be transmitted via the fecal-oral transmission route and associated with gastrointestinal illnesses, but also inhalation, dermal, and ocular contact routes can be responsible for respiratory illnesses, rash, and eye ailments. Among these, gastrointestinal illnesses are often the prevalent occurring pathologies compared to other illnesses, in beach recreational exposure, due to elevated contamination by fecal indicator bacteria (FIB), including fecal coliforms, *Escherichia coli*, and enterococci of human and animal gut origin, transported to the sand via water or directly spread by animals [1,2,3].

Urban waters can harbor high levels of FIB, suggesting that it may be an important source of contamination for beach sands [4,5].

Poultry fecal markers were detected which correlated with the presence of potential human pathogens, showing that disposal of contaminated poultry litters by land application can deliver FIB and other pathogens into receiving waters via runoff [6].

Human-associated microbial source tracking (MST) markers were reported in urban estuary, evidencing the significant impact of urban sprawl in rapidly growing countries in the contamination of coastal environments by waterborne pathogens, posing a critical risk to ecosystem and human health [7].

An association between FIB and human viruses was detected by MST analysis on California coastal beaches, showing that human sewage accounts, at least partially, for the degradation of water and beach quality, possibly impacting on human health risk [8].

However, while water quality is regulated by FIB enumeration, FIB/microbiological testing is not performed in the sand, thus providing inadequate information about contamination sources and associated health risk in beaches. Some studies regarding the analysis of sand contamination are available in the scientific literature, reporting the application of the MST method as a useful tool to assess beach sand contamination [1]. Besides, it was observed that high similarity in sand and water from the same site likely reflects anthropogenic contamination [9]. In addition, high FIB levels were reported in the sand of Azorean beaches, determining the contamination biological source by MST analyses of human and animal markers [1]; based on this, remediation measures were promptly implemented, including sand removal and spraying it with chlorine, to restore the beach sand quality. Overall, available studies support the need to perform community-based molecular studies to identify sources and potential impact of microbial pollution in the environment.

Being the beaches of the northern Adriatic Sea in Italy in close contact with water estuaries of two large rivers (i.e., Po and Reno rivers), likely collecting pollutants from many animal farms and urban cities along their ways, the aim of this study was to assess the level of microbial contamination in the sand of a beach located between the two river estuaries. To this aim, a combined approach was used for sand samples analysis, including simultaneous usage of conventional culture-based microbiological methods and molecular Whole Genome Sequencing (WGS) profiling. In addition, despite some studies have characterized the drug resistance of specific bacterial and fungal genera inhabiting the sand [10,11,12,13], a systematic investigation of the resistome of the whole sand microbial population is still lacking. Consistent with this, we also characterized the resistome of the sand microbial population by microarray, to add pieces of information about the drug resistance of the contaminating microbes. This approach, compared to those reported so far, allowed for the first time to obtain a comprehensive picture of the microbial community of the beach sand, together with a concurrent direct quantitation of the risk indicator microbes, needful for confrontation with legal references.

Last, based on the existing data, it appears that effective and safe methods for sand decontamination are needed. Reported remediation measures include sand removal and treatment by chlorine, to restore the biosafety sand quality [1]. Besides, quicklime used at 10–20% concentration (*w*/*v*) was reported as an effective treatment for highly contaminated sludges from wastewater plants [14]. Based on these observations, we tested the potential use of quicklime as a possible rapid sand decontamination method, by using low concentrations of quicklime (1–3%, *w*/*w*) mixed with sand, and analyzing over time its impact on the whole beach sand microbial population, as well on the fraction of potential human pathogens and on the environmental microbes.

## 2. Materials and Methods

### 2.1. Design of the Study

The study was conducted on sand samples derived from a beach of the North Adriatic sea (Cesenatico, Forlì-Cesena, Italy) and was subdivided in three consecutive phases: (1) preliminary analysis, to evaluate the microbial contamination of sand specimens collected from different sites and depths of the beach, which were analyzed by microbiological and molecular methods; (2) in vitro assays, to evaluate the antibacterial and antiviral ability of quicklime (calcium oxide, CaO) on the collected sand; (3) last, based on results obtained in previous phases, studies were performed on field, to test the antimicrobial activity of CaO in a controlled and supervised field, and to verify in real-life conditions the reproducibility of the results obtained in vitro. In these studies, the sand was mixed with different concentrations of CaO and the residual microbial population was assessed after different times of contact.

### 2.2. Beach Sand Sampling

For all the phases of the study, untreated and treated sand samples were collected from the beach by trained operators using sterile bags. Specifically, beach sites in the backshore, not experiencing periodic wetting and drying by the sea water, at 70 m from the sea water and at different depths (surface and 20 cm of depth) were collected, in order to assess their level/type of microbial contamination. Bags containing sand samples were hermetically closed, put in refrigerated boxes (2–8 °C), and transported within 4 h at the laboratory, where they were immediately processed. Sampling was performed from November 2020 to June 2021.

### 2.3. Microbiological Analyses

Culture-based microbiological analyses were performed on sand samples to evidence and quantify the presence of bacteria and fungi including potential human pathogens. Briefly, 5 g of each sand samples were placed in a 50 mL sterile tube, mixed with 30 mL of Tryptic soy broth (TSB, Biolife, Milan, Italy), and shaken by vortex for 1 min. The sand particulate was allowed to settle for 2 min and the supernatant was carefully removed without disturbing the sedimented particulate, which was discarded. The supernatant was centrifuged at 3200× *g* for 20 min to collect microbial pellets that were suspended in 2 mL of TSB each. Aliquots of 100 µL were serially diluted in sterile 0.9% NaCl and finally seeded in duplicate on agar plates containing general or selective microbial media. The following media (all from Biolife, Milan, Italy) were used: Tryptic soy agar (TSA), for total aerobes; Mannitol-salt agar (MSA), selective for *Staphylococcus* spp.; MacConkey agar (MCA), selective for *Enterobacteriaceae*; Cetrimide agar (CA), selective for *Pseudomonas* spp.; Bile Esculin Agar (BEA), selective for *Enterococcus* spp.; Sabouraud dextrose agar (SDA), selective for mycetes; Columbia blood agar (CBA), for anaerobes growth. The plates were incubated in specific conditions, depending on the microorganism type, as previously described [15,16]. In short, general and selective bacterial media were incubated at 37 °C for 24 or 48 h, respectively; CBA plates were incubated at 37 °C for 48 h in anaerobic jars (Anaerogen Systems, Thermo-Fisher Scientific Inc); and mycetes were incubated at 25 °C for 72 h. At the end of incubation time, grown Colony Forming Units (CFUs) were enumerated on duplicate plates seeded with serial dilutions (10^−1^ to 10^−4^) of the original samples. *Staphylococcus* spp. identification, including *S. aureus*, was performed by API Staph (Bio Merieux, Inc., Durham, NC, USA), as previously described [15,17].

### 2.4. Whole Genome Sequencing (WGS) Analyses

The sand samples processed as described for microbiological analyses, to obtain a microbial suspension in TSB (2 mL/sample) corresponding to 5 g of sand. The 2 mL suspension was then subdivided in two aliquots, which were further centrifuged at 14,000× *g* for 10 min, to collect also sub-microbial viral particles. Obtained pellets were frozen and kept at −80 °C until use. The total DNA was extracted from pellets by using the Dneasy Power Soil Pro kit (Qiagen, Hilden, Germany), following the manufacturer’s instructions. Extracted DNA was checked and quantified by spectrophotometric reading at 260/280 nm, using a nanodrop (Thermo Scientific, Milan, Italy). Prior to downstream analyses, the amplificability of extracted DNA was checked by PCR amplification of housekeeping bacterial and fungal genes, respectively performed by bacterial 16S rRNA gene (pan bacterial PCR, panB), and fungal ITS gene (pan fungal PCR, panF) PCR, as previously described [15,18,19].

WGS analyses were performed on 100 ng of extracted and checked DNA, by the NGS Service of the University of Ferrara (Department of Morphology, Surgery and Experimental Medicine, University of Ferrara, Ferrara, Italy), who carried out library preparation, sequencing, and taxonomic analysis, as previously described [18]. In short, WGS libraries were prepared using NEBNext^®^ Fast DNA Fragmentation and Library Prep Kit for Ion TorrentTM (ThermoFisher Scientific, Milan, Italy), following the manufacturer’s protocol. Samples were then sequenced by using the Ion Gene Studio S5 System (ThermoFisher Scientific, Milan, Italy). The taxonomic assignment has been performed using Kraken2 (Pubmed ID: 24580807) and a database consisting of archaea, bacteria, fungi, protozoa, and viruses. Raw sequencing data and bioinformatics analyses have been deposited in the European Nucleotide Archive (ENA) website (accession number PRJEB61323). A total of 48 samples were analyzed, including both original sand samples used in in vitro preliminary studies and the samples derived from subsequent on-field tests.

### 2.5. Microarray Analyses

Quantitative real-time (qPCR microarray) analyses were performed to further characterize the sand microbial population with regard to the presence of specific species and of genes conferring drug-resistance. For specific species identification, 1 µg of DNA extracted from sand samples was analyzed by the Water Analysis Microbial DNA qPCR Array Microbial Profiling (Qiagen, Hilden, Germany), able to detected and quantify simultaneously 45 bacterial species frequently assessed as markers of water microbial contamination, following the manufacturer’s instructions. The sensitivity declared by the manufacturer for each measured parameter is comprised between 20 and 100 copies per sample. The results, expressed as dCt, were elaborated by the free data analysis Qiagen software to obtain relative quantitation with respect to negative control (NTC).

For the analysis of the resistome of the sand microbial community, 5 g of sand were first put in 10 mL of TSB and incubated under mild agitation at 37 °C for 24 h, to obtain a controlled microbial amplification. The obtained microbial suspension was then centrifuged at 12,000× *g* for 5 min, and the total DNA was extracted from the pelletized microbes by the Exgene Cell SV mini kit (Gene All, Seul, Republic of Korea), following the manufacturer’s instructions. One µg of extracted DNA was then analyzed using the Microbial DNA qPCR Array for Antibiotic Resistance Genes (Qiagen, Hilden, Germany), allowing the simultaneous detection and quantification of 84 genes coding for antimicrobial resistance (AMR), as previously described [15,17,19,20,21,22].

### 2.6. In Vitro Evaluation of CaO Action

The decontaminating potential of CaO on the collected beach sand samples was first assessed in vitro. Briefly, 10 g of sampled sand were put in sterile 50 mL tubes and mixed with 1, 2.5, and 5% (*w*/*w*) of CaO, in the presence of 15% (*w*/*w*) of sterile bidistilled water, needed to allow a proper action of CaO. Control samples received only water. After 1, 2, 24, and 48 h of contact, 1 g aliquot of sand was collected, put in a new sterile tube with 2 mL of sterile physiological solution (NaCl 0.9%), vortexed for 30 s and let to settle for 2 min at room temperature. The pH value of supernatant was then evaluated and 100 µL aliquots were seeded in duplicate on TSA plates and incubated for 24–48 h at 37 °C. Residual CFUs were enumerated at the end of the incubation.

### 2.7. Antiviral CaO Activity Assays

To assess the CaO antiviral activity, the following virus strains and target cells were used. Modified vaccinia virus Ankara (MVA) (ATCC VR-1508), Human alpha-coronavirus 229E (hCoV-229E) (ATCC VR-740), and Enterovirus 71 (EV71) (ATCC VR-784). The indicated viruses were expanded and titrated using respectively the BHK-21 fibroblast cell line (ATCC CCL-10), the MRC-5 fibroblast cell line (ATCC CCL-171), and the Vero-E6 fibroblast cell line (ATCC CRL-1586). MRC5 cells were cultured in Eagle Minimal Essential Medium Eagle (EMEM)(Gibco, Grand Island, NY, USA), whereas Vero-E6 and BHK-21 cell lines were expanded in Dulbecco’s Modified Eagle’s Medium (Gibco, Grand Island, NY, USA). Cells were grown at 37 °C + 5% CO_2_, in the appropriate complete culture medium, consisting of EMEM or DMEM supplemented with 10% fetal bovine serum (FBS), 2 mM L-Glutamine, 100 U/mL penicillin, and 100 µg/mL streptomycin (complete EMEM/DMEM-10) (Gibco, Grand Island, NY, USA).

Viruses’ stock preparation and titration were performed as follows. Monolayers of target cells at 90% confluence were infected and incubated at 37 °C + 5% CO_2_ in culture medium additioned with 2% FBS (EMEM/DMEM-2), until appearance of an evident cytopathic effect (CPE), affecting >80% of cultured cells (2 days for MVA, 5 days for EV71, 7 days for hCoV-229E). After the appropriate incubation times, cells and culture supernatants were collected, cellular fractions were lysed by 3 cycles of freezing/thawing in liquid nitrogen and 37 °C water bath, with 30 s pulse-vortex intervals, and cell lysates were added to culture supernatant. Virus stocks were obtained by centrifugation at 20,000× *g* for 45 min at 4 °C, and resuspension of viral pellets in 1 mL of PBS + 1% bovine serum albumin (BSA), then 0.1 mL aliquots were immediately frozen in liquid nitrogen and kept at −80 °C until use. Viral titrations were performed in 96-well plates, by infecting sextuplicate samples of the appropriate target cells with serial dilutions of viral inocula, and assessing the 50% tissue culture infectious dose (TCID_50_) per mL by the standard Spearman-Karber method, as previously described [23]. All virus stocks used for the subsequent assays of CaO antiviral activity ranged from 10^7.8^ to 10^8.2^ TCID_50_/mL, as quantified by the Spearman-Karber method.

The antiviral activity of CaO was evaluated first on non-porous hard plastic surfaces and then in sand. In the first assays, 15 µL of virus stock suspension (corresponding to 1.5 × 10^5.8^–1.5 × 10^6.2^ TCID_50_/mL, depending on the virus type) were seeded in each well of a 48-well plate and an amount of powder corresponding to 85 µL of volume and containing 1, 2, or 3% of CaO was added immediately on the 15 µL of virus suspension (providing 15% final humidity). Talcum powder was used as a negative e control and to adjust CaO concentration. Ethanol 70% in water (70% EtOH) was also included as positive control. Control of CaO cytotoxicity was performed by adding the same amount of CaO to 15 µL of culture medium +2% FBS. All samples were incubated at room temperature for 1 h, then the content of each well was collected in 1.5 mL of cold EMEM/DMEM-2, filtered by 0.45 µm sterile filters, and serially diluted (1:10 dilutions) in cold EMEM/DMEM-2. The residual viral titre of each serial dilution was assessed by Spearman-Karber method.

In the assays performed in sand matrix, 1 g of sand was put in a 50 mL sterile tube and artificially contaminated with 200 µL of virus suspension containing 1.5 × 10^5.8^–1.5 × 10^6.2^ TCID_50_, depending on virus type. Virus-contaminated sand samples were immediately pulse-vortexed for 30 + 30 s to distribute uniformly the virus, then they were mixed with amounts of 1–2–3% of CaO (*w*/*w*, with respect to the sand volume), and pulse-vortexed for further 30 s. Untreated and 70% EtOH treated sand samples were included as negative and positive control, respectively. Control of CaO cytotoxicity was performed by adding 200 µL of EMEM/DMEM-2 instead of virus. After 1 h of incubation at room temperature, the residual virus was recovered from the sand by adding 2 mL of cold EMEM/DMEM without FBS (to avoid foaming during processing) and pulse-vortex for 30 + 30 s. The sand fraction was allowed to settle at the bottom of the tube for 2 min, then the supernatant was collected, filtered (0.45 µm) and serially diluted EMEM/DMEM-2. Residual viral titre in each serial dilution was assessed by the Spearman-Karber method, as described for surface test. Each condition was assayed in three independent assays with duplicate samples.

### 2.8. Evaluation of CaO Action on Field

Based on the results obtained in vitro, the potential of CaO use as a sand decontaminant was assessed on field. Two types of assays were conducted sequentially, first in controlled field and then in supervised field. Specifically, the study in controlled field was performed on 1 m^3^ of sand transported in a controlled area close to the laboratory. Based on in vitro results, 1, 2, and 3% of CaO (*w*/*w*) were tested. After careful mixing of CaO and sand samples were collected after 1 and 2 h, and after 1, 2, 7 and 14 days of contact. At each timepoint, 5 g of sand were collected, put in 30 mL of sterile 0.9% NaCl, vortexed for 1 min, and let to settle for 2 min; 0.3 mL aliquots of supernatant (corresponding to 0.05 g of sand) were then seeded in duplicate on TSA and CBA plates and incubated at 37 °C in aerobic and anaerobic conditions, respectively, to enumerate aerobes and anaerobes respective CFUs.

A further study was performed in a supervised, inaccessible area of the beach from which the sand was originally collected. One m^3^ of sand was treated by mixing with 1–2–3% CaO (*w*/*w*), and samples were collected after 1 and 2 h, and 1, 2, 7, 14, 30, 47, 63, and 100 days of contact. At each timepoint, 5 g sand samples were collected and processed as described for the study in the controlled field, and aerobes and anaerobes CFUs were enumerated. Untreated sand and sand treated with 3% (*w*/*w*) of CaCO_3_, which is the inactive compound generated by exhausted CaO, were included as controls.

### 2.9. Statistical Analyses

Statistical analyses were performed using the GraphPad Prism software. Parametric Student’s t test was used to compare groups, assuming as statistically significant a *p* value ≤ 0.05. For microarray data, the Bonferroni correction for multiple comparisons was applied to the detected Student’s *t* test value, and a p_c_ value ≤ 0.05 was considered significant. Statistical analyses for sequencing results were performed with Agilent GeneSpring GX v11.5 software (Agilent Technologies, Santa Clara, CA, USA) and R (R 2019, R Core Team, available as a free software at https://www.r-project.org/, accessed on 25 May 2023). Data were expressed as relative abundance of each taxonomic unit at phylum, genus, or species level. Alpha-diversity was used to describe the microbiome diversity between untreated or treated sand samples, and was obtained by measuring the Shannon H′ diversity index.

## 3. Results

### 3.1. Characterization of Beach Sand Microbiame

To characterize the basal microbial population in the sand of the sampled beach (Cesenatico, Forlì-Cesena, Italy), 8 sand samples were collected from the backshore area of the beach, at 70 m from the sea water, four from surface and four from 20 cm of depth. Each sample was immediately put in a sterile bag, refrigerated, and processed within 4 h from the withdrawal. All collected samples were analyzed by both conventional culture-based microbiological methods and molecular methods (WGS and qPCR microarray), to obtain a comprehensive profile of the microbial community colonizing the beach sand.

**Figure 1:**
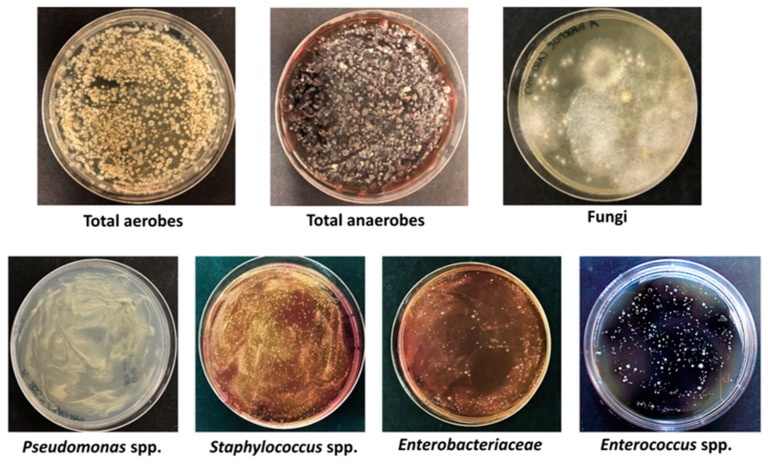
Bacterial and fungal microbes detected in sand by culture on agar media. Total aerobes, total anaerobes, fungi, *Pseudomonas* spp., *Staphylococcus* spp., *Enterobacteriaceae*, and *Enterococcus* spp. CFUs were respectively enumerated on TSA, CBA, SDA, CA, MSA, MCA, and BAE plates, after appropriate incubation. Depicted results are indicative of what obtained in all collected samples, after seeding 100 µL of the solution obtained from 0.25 g of sand.

For microbiological analyses, 0.1 mL aliquots of microbial suspension, corresponding to 0.25 g of sand, were serially diluted in 0.9% NaCl and seeded in duplicate in general and selective media, including Tryptic Soy Agar (TSA), for the count of total microbes; Mannitol Salt Agar (MSA), selective for *Staphylococcus* spp.; McConkey Agar (MCA), selective for *Enterobacteriaceae*; Cetrimide agar (CA), selective for *Pseudomonas* spp.; Bile Esculin Agar (BEA), selective for *Enterococcus* spp.; Sabouraud dextrose agar (SDA), selective for mycetes; and Columbia blood agar (CBA), used for the growth of exigent anaerobes. After incubation in the appropriate conditions, CFUs were enumerated, evidencing the presence of >10^4^ CFU/g of sand for all searched microorganisms (Figure 1).

Among aerobic bacteria, *Pseudomonas* genus was the most prevalent (>10^5^ CFU/g), followed by *Staphylococcus* (>10^4^/g), *Enterococcus* spp. (>10^3^ CFU/g), and *Enterobacteriaceae* family (>10^3^ CFU/g). The anaerobic *Clostridium* genus was also abundant (>10^4^ CFU/g). Besides bacteria, fungi including yeasts and moulds were also detectable at fair level (Table 1).

**Figure 2:**
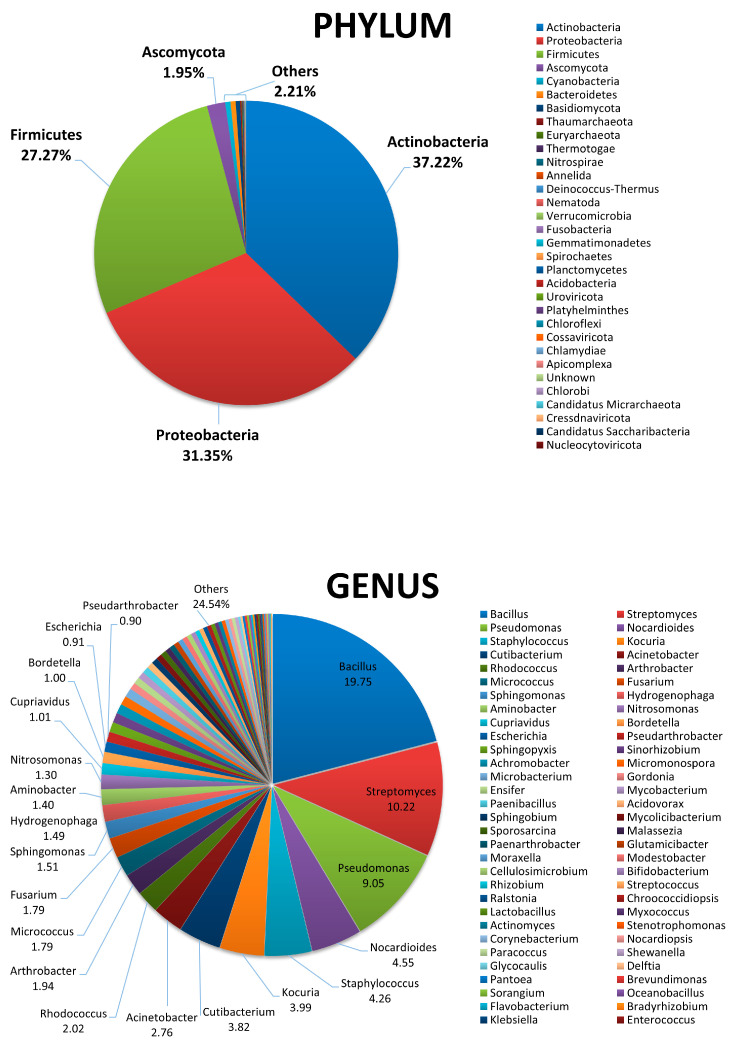
Phylum and genus composition of the beach sand microbiome, as detected by WGS analysis. Results are expressed as percentages of relative abundance.

To obtain a comprehensive picture of the whole sand microbiome, including unsearched and non-culturable microbes, the same sand samples were analyzed by WGS, able to provide detailed characterization of the entire microbial community, including eukaryotes and viruses. By WGS analysis, the taxonomy and community composition of sand microbiome showed the prevalence of bacteria of *Actinobacteria*, *Proteobacteria*, and *Firmicutes* phyla, with relative abundance values of 37.22%, 31.35% and 27.27%, respectively (Figure 2). The only other phylum showing a relative abundance > 1% was represented by the eukaryotic fungi *Ascomycota* (1.95%), whereas all the other detected phyla showed values of relative abundance < 1%. Among them, the more represented included bacteria, mycetes and archaea, specifically *Cyanobacteria* (0.56%), *Bacteroidetes* (0.55%), *Basidiomycota* (0.46%), *Thaumarcheota* (0.14%), and *Euryarcheota* (0.08%). *Anellida*, *Nematoda*, and *Platyhelminthes* metazoans were also detectable, as well as DNA viruses (*Cossaviricota*, *Uroviricota*, *Cressdnaviricota*, *Nucleocytoviricota*), representing together the 0.01% of the total detected phyla. Due to the extraction method used, RNA viruses could not be detected.

At the genus level, the results showed the prevalence of bacteria belonging to the *Bacillus* genus (19.75% relative abundance), followed by *Streptomyces* (10.22%) and *Pseudomonas* spp. (9.05%). The top twenty microbial genera in the sand microbiome also included *Nocardioides* (4.55%), *Staphylococcus* (4.26%), *Kokuria* (3.99%), *Cutibacterium* (3.82%), *Acinetobacter* (2.76%), *Rhodococcus* (2.02%), *Arthrobacter* (1.94%), *Micrococcus* (1.79%), *Fusarium* (1.79%), *Sphingomonas* (1.51%), *Hydrogenophaga* (1.49%), *Aminobacter* (1.40%), *Nitrosomonas* (1.30%), *Cupriavidus* (1.01%), *Bordetella* (1.00%), *Escherichia* (0.81%), and *Pseudoarthrobacter* (0.90%).

At the species level, the distribution mirrored that evidenced by genus analysis, showing the prevalence of *Priesta megaterium* of the *Bacillus* genus (11.75% of relative abundance), followed by *Streptomyces alfalfae* (7.92%), *Pseudomonas putida* (5.84%), *Kokuria rosea* (3.76%), *Cutibacterium acnes* (3.72%), *Acinetobacter guillouiae* (2.43%), *Micrococcus luteus* (1.80%), *Nocardioides* CF8 (1.80%), *Bacillus cereus* (1.76%), *Fusarium oxysporum* (1.70%), *Priestia flexa* (1.70%), *Staphylococcus epidermidis* (1.63%), *Nocardioides dokdonensis* (1.50%), *Hydrogenophaga* PBC (1.41%), *Nitrosomonas ureae* (1.23%), *Bacillus thurigensis* (1.10%), *Staphylococcus hominis* (0.97%), *Pseudomonas chloroaphis* (0.97%), *Arthrobacter crystallopoietis* (0.92%), and *Escherichia coli* (0.91%).

Since the WGS results evidenced the presence of several microbes of putative human/animal origin in the sand, though at lower prevalence compared to environmental microorganisms, we wanted to profile in more detail the eventual presence of potential pathogens scarcely represented in the total sand microbiome. To this aim, the same samples were analyzed by a qPCR microarray able to simultaneously detecting 45 bacteria frequently assessed as markers of water microbial contamination. The results confirmed the presence of several potential human pathogens, including *Campylobacter*, *Clostridium*, *Enterococcus*, *Shigella*, *Streptococcus*, *Vibrio*, and *Yersinia* species (Table 2). The genes coding for the virulence factors *eae* (intimin adherence protein) and *stx2A* (Shiga-like toxin II), present in the pathogenic enterohemorrhagic strains of *Escherichia coli* [24,25], were also detected.

**Figure 3:**
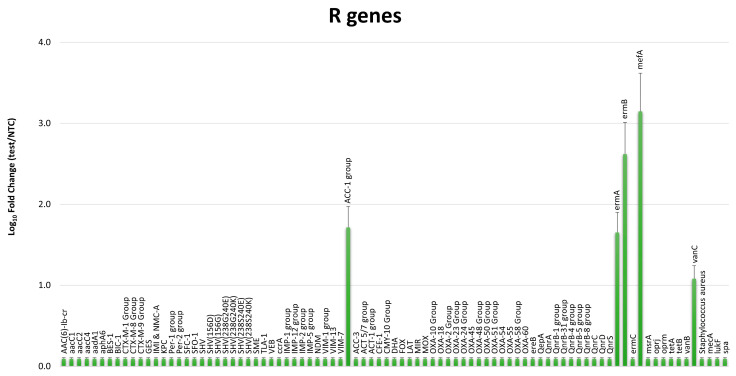
Resistome characterization of the sand microbiome. Results are expressed as mean value ± S.D. of Log_10_ fold changes for each resistance (R) gene compared to negative controls (NTC).

The resistome of the sand microbiome was also analyzed, to further characterize the drug-resistance features of the beach sand microbial population, by using a qPCR microarray able to simultaneously detect and quantify 84 bacterial genes coding for antibiotic resistance. The collected data (Figure 3) evidenced the presence of antimicrobial resistance determinants in the sand microbiome, including genes of *AAC-1* group, coding for acetyltransferases able to confer resistance to aminoglycoside by enzymatic modification of the antibiotic; *ermA*, inducible by erythromycin and conferring resistance against macrolides, streptogramin, and lincosamides, and frequently detected in *Enterococcus* and *Staphylococcus* genera; *ermB*, conferring resistance to macrolides similar to *ermA*, and most frequently detected in *Enterococcus faecium*; *mefA*, also associated with the resistance to macrolides by the expression of efflux-protein and detectable in *Streptococcus* and *Enterococcus* spp.; *vanC*, a D-Ala.D-Ala ligase homolog that synthesizes D-Ala-D-Ser as an alternative substrate for peptidoglycan synthesis, reducing the binding affinity of vancomycin antibiotic, mostly prevalent in Enterococci and particularly in *E. gallinarum*.

### 3.2. Effect of Quicklime (CaO) on Sand Microbial Contamination

#### 3.2.1. In Vitro Studies

**Figure 4:**
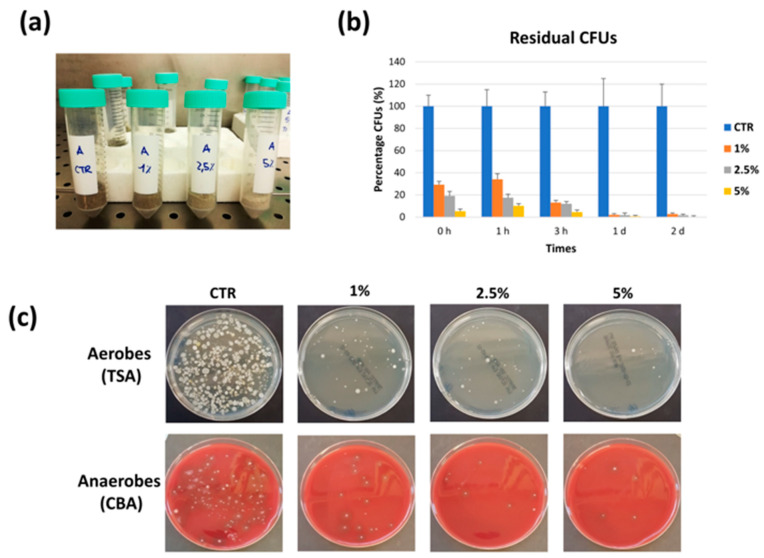
In vitro CaO assays. Beach sand samples were mixed with increasing amounts of CaO (1, 2.5, and 5%, *w*/*w*) and 15% water, or with water alone (control, CTR) and incubated at room temperature for the indicated times. (**a**) Whitening effect of CaO on sand samples. (**b**) Decrease of CFU number in treated sand samples. Results are expressed as percentage of CFU number in CaO-treated samples compared to controls, representing 100%, and derive from duplicate samples in two independent assays. (**c**) Decrease of aerobic and anaerobic microbial population in treated sand samples. Pictures were taken after 1 h of contact and are representative of all the analyzed samples.

Based on the detection of several potential pathogens in the sand microbiome, we assessed the potential of CaO for beach sand remediation purposes. First, CaO activity was assayed in vitro, by mixing 10 g of sampled sand with 1, 2.5, and 5% CaO (*w*/*w*), in the presence of 15% of water, to allow optimal CaO action. Residual microbial load was then assessed after 1, 2, 24, and 48 h of contact, by CFU counting. Sand-CaO mixing resulted in immediate exothermic reaction and whitening action on treated sand (Figure 4a). This early effect was accompanied by a dose-dependent and immediate decontaminating action, which at 0 h (immediately after mixing) reduced the original microbial CFUs of 70%, 80%, and 94% in samples treated with 1%, 2.5%, and 5% of CaO, respectively. After 1 and 3 h, the microbial population appeared further decreased in CaO-treated, reaching −87% with 1% CaO, −88% with 2% CaO, and −96% with 5% CaO, compared to controls. The decontamination was even more evident at 1 and 2 days of contact, when between 98% and >99% of the control CFUs were disappeared, with all tested CaO concentrations.

Monitoring samples’ pH evidenced an increase of pH toward basicity in CaO-treated vs. control samples (Table 3). After 1 h of contact, pH values increased from pH 9.38 of untreated controls to pH > 12 in CaO-treated samples. pH values then remained higher than controls till the end of the experimentation (2 days), although it tended to decrease in a concentration-dependent mode.

The CaO activity against viruses was also assessed. Due to the very low concentration of measurable viruses in the original sand samples, the antiviral action of CaO was tested in artificially contaminated samples, by using Modified vaccinia virus Ankara (MVA, *Poxviridae* family) and Enterovirus 71 (EV71, *Picornaviridae* family), as viral contaminants. MVA and EV71 were chosen as respectively representative of the most resistant enveloped virus and of highly resistant naked viruses which could persist in beach sand in real life conditions.

**Figure 5:**
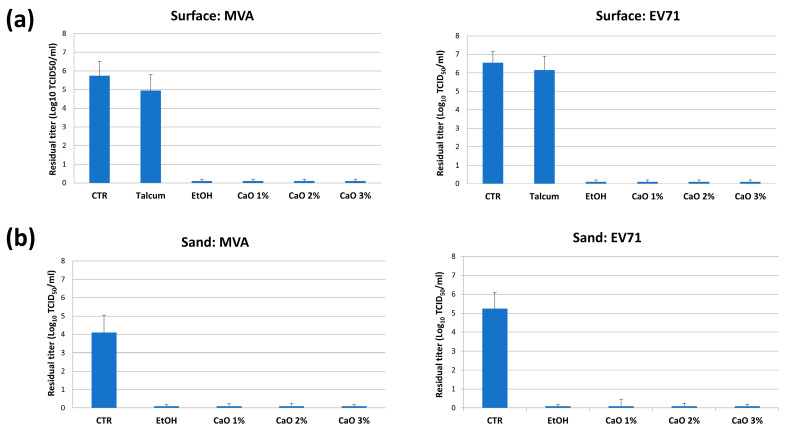
In vitro antiviral activity of CaO. (**a**) Results of surface tests, performed in sterile plastic plates. (**b**) Results of tests performed in sand matrix. Results refer to duplicate samples from three independent assays, and are expressed as mean virus titer ± S.D.

First, the CaO action was assayed on non-porous hard plastic surfaces, contaminated with 15 µL-aliquots of viral suspension containing >10^5^ TCID_50_ and treated by adding 1–2–3% of CaO powder in a final volume of 100 µL. Talcum powder and 70% Ethanol (EtOH) were included in the assays as negative and positive control. After 1 h of incubation at room temperature, samples were collected in 1.5 mL of cold EMEM/DMEM-2 and the residual virus titer was measured by the Spearman-Karber method. The results (Figure 5) showed that any CaO concentration was able to inactivated totally both viruses in 1 h of contact, similarly to what observed with 70% EtOH, whereas talcum powder did not induce virus titer alterations.

Based on these results, CaO action was tested in sand matrix, by contaminating 1 g of sand with >10^5^ TCID_50_ of MVA or EV-71. Contaminated sand was stirred to distribute uniformly the virus, then immediately mixed with 1%, 2%, 3% (*w*/*w*) of CaO. Talcum powder and 70% EtOH were included as negative and positive controls. After 1 h of contact at room temperature, the Spearman-Karber titration of residual virus evidenced full inactivation of both target viruses also in sand matrix, at all tested CaO concentrations (Figure 5b).

#### 3.2.2. On Field Studies

**Figure 6:**
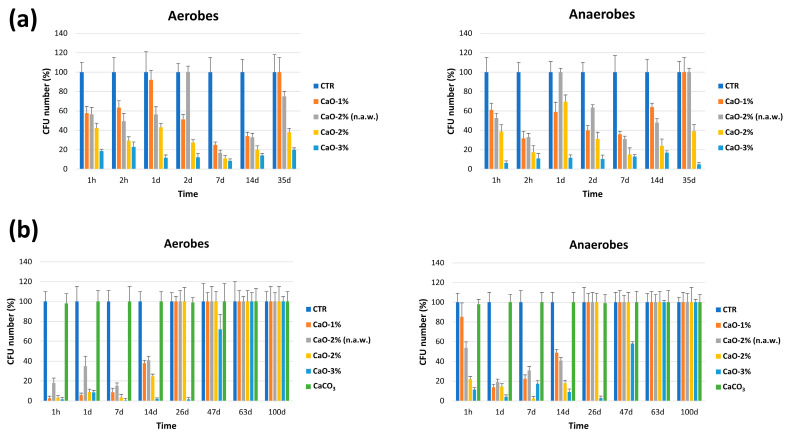
CaO antimicrobial activity on field. (**a**) Controlled field study; 1 m^3^ of sand was mixed with the indicated CaO concentrations (*w*/*w*) in the presence of 15% water (*w*/*w*); 2% CaO was also tested with no added water (n.a.w.); untreated sand was used as a control (CTR). (**b**) Supervised field study; 1 m^3^ of sand was mixed with the indicated CaO concentrations (*w*/*w*) in the presence of 15% water (*w*/*w*); 2% CaO was also tested with no added water (n.a.w.); untreated (CTR) and CaCO_3_-treated sand were also included as controls. Results are expressed as percentage of reduction in treated sand compared to untreated controls, taken as 100% value.

Based on in vitro results, the CaO decontaminating action was analyzed on field. Controlled field assays were performed first, in a protected area close to the laboratory, where 1 m^3^ of sand was mixed with 1, 2, and 3% (*w*/*w*) of CaO in the presence of 15% (*w*/*w*) of water. In addition, 2% of CaO (*w*/*w*) was also tested in the presence of natural humidity alone (5%, as measured by hygroscope on site). Samples corresponding to 5 g of sand were collected after 1, 2 h, and 1, 2, 7, 14, and 35 days of contact. Each sample was mixed by agitation with 30 mL of sterile physiological solution, and 0.3 mL supernatant aliquots (corresponding to 0.05 g of sand) were seeded on TSA and CBA plates, used respectively to enumerate total aerobic and anaerobic CFUs. The results (Figure 6a) showed that CaO significantly diminished the level of aerobic and anaerobic microbes compared to what detected in untreated controls (respectively 1064 ± 148 CFU/sample and 257 ± 101 CFU/sample, median value ± S.D. of all timepoints). CaO action was dose-dependent, and the same CaO concentration (2%) action was lower in the absence of added water. 1–2% CaO concentrations were not anymore active at 35 days, whereas 3% CaO maintained the decontamination ability till the end of experimentation.

Decrease percentages, expressed as median values of all timepoints compared to controls, corresponded to −49% for 1% CaO (542 ± 150 CFU/sample), −65% for 2% CaO with no added water (371 ±181 CFU/sample), −71% for 2% CaO (306 ± 95 CFU/sample), and −90% for 3% CaO (102 ± 14 CFU/sample). Total anaerobes decreased as well, compared to controls, in a superimposable way.

Based on these evidences, a study in supervised field was performed in a inaccessible area of the beach, from which the sand samples were collected for all the previous assays. A portion of sand corresponding to 1 m^3^ volume was stirred with 1–2–3% of CaO and samples were collected at 1 h and 1, 7, 14, 26, 47, 63, and 100 days. Controls included untreated sand and sand treated with 3% of calcium carbonate (CaCO_3_; *w*/*w*), which is the inactive compound generated by exhausted CaO. The results obtained in the open supervised field were very similar to those obtained in controlled field (Figure 6b), except for a slightly earlier loss of activity of CaO: specifically, 1–2% CaO became inactive at 26 days, while 3% CaO was still effective at 47 days (28% decrease of aerobes and 90% decrease of anaerobes) and became inactive at 63 days. No alterations were detectable at any time in the sand treated with 3% CaCO_3_, with respect to controls, confirming the lack of antimicrobial activity of this compound.

Monitoring of pH values confirmed what observed in vitro, showing a pH increase in CaO-treated sand compared to control in just 1 h of contact (Table 4). The pH increase ranged from 2.02 to 3.37 units and was detectable at all CaO concentrations, from 1 h to 14 days after contact. At later timepoints (26 and 47 days), increased pH values were still detectable only in the 3% CaO-treated sand. No alterations were observed at 63–100 days at any CaO concentrations or at any time in CaCO_3_-treated sand, compared to controls.

**Figure 7:**
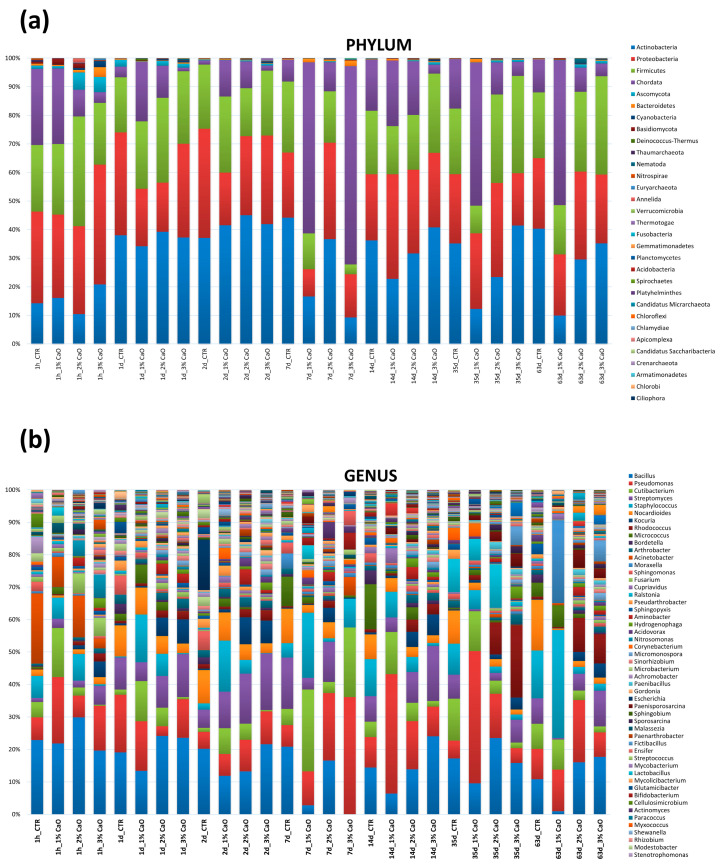
Microbiome profiling of control and CaO-treated sand obtained by WGS. (**a**) Composition of each sample at phylum level. (**b**) Composition of each sample at genus level. The results are expressed as relative abundance values of phyla and genera in each collected sample.

To profile in detail CaO-treated sand compared to controls, on-field samples were also analyzed by WGS. Based on the microbiological data, samples collected at 1 h, and 1, 7, 14, 26, 47, and 63 days were analyzed (Figure 7). Overall, a remarkable fluctuation in the relative abundance of sand microbiome phyla and genera was observed during the study period in controls, perhaps due to the exposure of sand to environmental conditions, which impacted on the microbial population residing in the beach sand. However, no significant variations were observed at any time in CaO-treated samples compared to controls, despite the evident decrease of total contamination demonstrated by CFU count analyses. Thus, rather than substantial changes in the microbiome composition, a quantitative decrease of the whole microbial population was observed, which was better evidenced by CFU counts.

**Figure 8:**
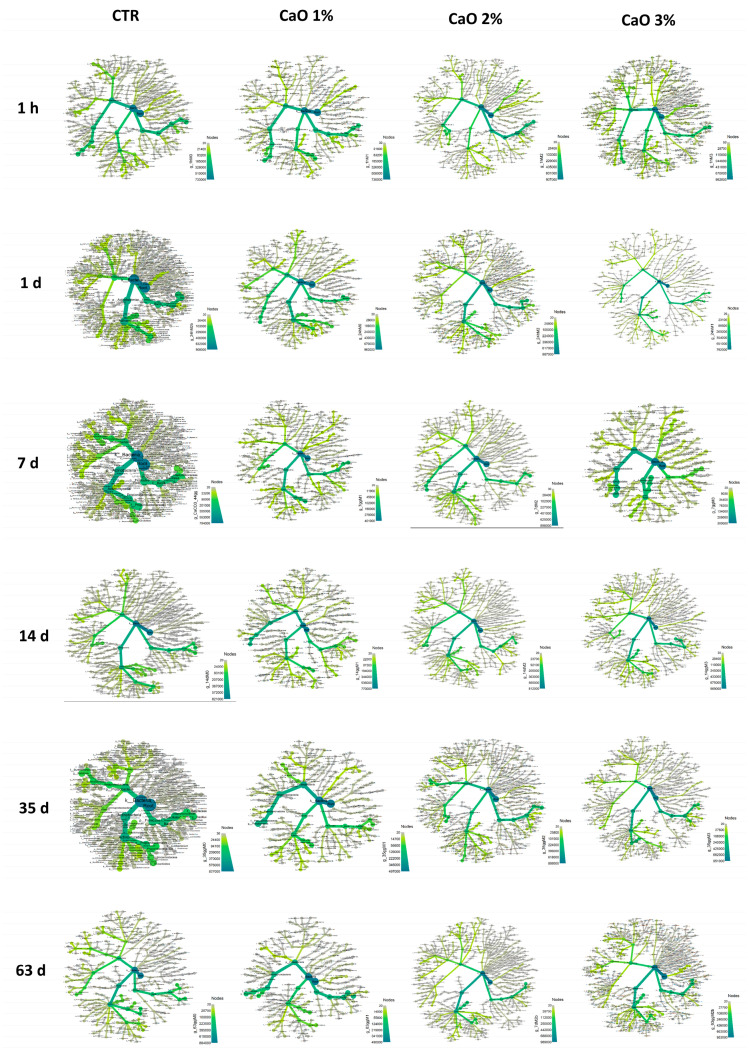
Heat trees representation of the relative abundance detected in untreated and CaO-treated beach samples at the indicated times. Phyla, classes, order, families, genera, and species are represented. Node label, taxon name; node size, number of operational taxonomic units (OTUs); node color, abundance of the indicated phylum/class/family/genus/species (from grey to green, as reported in the color scale).

To obtain a more precise representation of the quantitative and qualitative variations in control and treated sand samples, WGS data were elaborated to build heat-tree maps (Figure 8).

The heat-tree maps evidenced a CaO-induced decrease in the amount of almost all detected genera, compared to controls, starting from 1 day after treatment. At 1 h, in fact, despite the immediate decrease of viable bacteria observed by CFU enumeration, no alterations were seen at the DNA level, likely due to the persistence of bacterial DNA genomes, despite cell death. At 1 day, cell-free DNA and dead cells were instead likely degraded and the abundance of bacteria was apparently decreased, compared to untreated controls. Such differences were not further detectable at 35 days for CaO 1% and 2%, and at 63 days for CaO 3%, when the microbial population went back to the control values, showing no significant alterations compared to the population detected in untreated controls, and confirming the reversibility of the CaO effect.

To evidence in more detail any eventual significant variation in the CaO-treated sand compared to controls, the sand core species during the entire study period were identified and analyzed. The core sand microbiome included 37 bacterial species (Table 5), most of which were present also in CaO-treated samples, confirming the lack of substantial alterations of the sand microbiome composition following CaO treatment. However, a significant decrease in the quantity of some individual bacterial species was detected (*p* < 0.05), including *Cutibacterium acnes*, diverse *Nocardioides* species, *Priestia flexa*, *Sphingomonas koreensis*, *Pseudarthrobacter phenanthrenivorans*, *Pseudomonas aeruginosa* and *stutzeri*, *Bordetella petrii*, and *Microbacterium* sp. *Y-01*. Besides, other species were decreased, sometimes resulting virtually disappeared, but the differences were not statistically significant.

To further describe any effect of CaO on the composition of sand microbiome, WGS data were used to calculate the alpha-diversity values by measuring the Shannon H′ diversity index in collected sand samples, which expresses the species richness of each sample. The alpha-diversity values ranged from 8.86 to 13.72 in control samples (Table 6), and superimposable or non-significantly varied values were detected in all CaO-treated samples at most times. Significantly decreased values were only observed in 3% CaO-treated samples at 1, 2, 7, and 14 days after treatment, whereas no variations were observed at later times (35 and 63 days).

## 4. Discussion

Recreational waters have long been monitored for possible human pathogens, searching the presence and abundance of microbes of human or animal fecal origin, while microbial communities in the sand have received relatively little attention compared to those in water, and only recently the microbiological quality of the beach sand has begun to be tested as well [26]. Of note, recent studies report that microbial pathogens in beach sands are increasing and that not all are of fecal origin as initially identified [27]. Several human-associated microbes have been found in samples of beach sand, including bacteria, fungi, viruses, and parasites [9,26,27]. Besides typical fecal contaminants, other microbes have been frequently reported, whose abundance is tightly correlated with the number of people frequenting the beach, such as *Staphylococcus* spp. [27], potentially causing infections by entering the skin through cuts and scrapes [27]. Also the so-called flesh-eating bacteria have emerged recently, including common species belonging to *Streptococcus*, *Klebsiella*, *Clostridium*, *Escherichia*, *Staphylococcus* and *Aeromonas* genera, all capable of entering the skin through small cuts or lesions, and causing necrotizing fasciitis, a severe infection destroying soft tissues [27]. Fungal pathogens have also been reported in beach sand, including skin *Dermatophytes* such as *Trichophyton mentagrophytes* and *Trichophyton rubrum*, respectively the most common source of dermatomycosis in Europe and in the world, and opportunistic fungi such as *Aspergillus* and *Candida* spp. [27]. As well, viruses were detected in beach sand, including enteroviruses and reoviruses, associated with lung and gut infections, found in at least 23% of beach sand samples [27,28]. Parasites have also been detected, including *Toxocara canis* [27], which usually infects dogs but can also infect humans potentially inducing liver, eye, myocardium and lung diseases [27].

Thus, understanding beach microbial ecology appears important to foresee the possible impact of sand contamination on public health, and efforts to monitor the community of beach microbes and its changes could be crucial.

Consistent with this purpose, this study was aimed to characterize the microbiome of an Italian beach facing the Northern Adriatic Sea, to evaluate any eventual potential pathogenic implication and identify possible remediation actions.

The characterization of the sand microbiome, obtained by both culture-based and molecular analyses, showed the presence of >10^3^/g of *Enterobacteriaceae*, *Pseudomonas aeruginosa*, *Enterococci*, and *Staphylococci*. Considering that the maximum allowable level for fecal coliforms in marine water is 400 CFU/100 mL, and 105 CFU/mL for Enterococci, the levels detected in beach samples were worthy of attention. Notably, a recent WHO guideline indicates 60 CFU/g as the limit for intestinal Enterococci in beach sand [29]. Most Enterococci were likely of animal origin (*E. gallinarum* and *casseliflavus*), with only a minor part represented by *E. faecalis* and *faecium*, highlighting the impact of local animal farms on the composition of beach sand microbiome. Other human pathogens detected by qPCR microarray included *Arcobacter butzleri*, *Desulfovibrio desulfuricans*, *Morganella morganii*, *Shigella dysenteriae*, *Vibrio cholerae*, and *Yersinia enterocolitica*, these too probably related to the presence of several breedings in the region close to the sampled beach. Moreover, WGS profiling revealed the presence of many human potential opportunistic pathogens with significant relative abundance in sand microbiome, including *Kokuria rosea* (3.76%), a skin Gram-positive coccus implicated as an opportunistic pathogen [30]; *Cutibacterium acnes* (3.72%), a skin commensal potentially acting as an opportunistic pathogen [31]; *Micrococcus luteus* (1.80%), a normal inhabitant of human skin and oropharynx mucosa which can cause opportunistic infections [32]; *Staphylococcus epidermidis* and *hominis* (2.6% altogether), usual skin colonizers with important role in skin and multiorgan diseases [33]; and *Escherichia coli* (0.91%), a well-known gut colonizer with high pathogenic potential in many illnesses [34]. Other opportunistic pathogens often naturally occurring in water (*Pseudomonas*, *Acinetobacter*) were detected at low abundance. Fungi belonging to *Ascomycota* (1.95%) and *Basidiomycota* (0.46%) were also detected, although at a lesser extent compared to the dominance of bacteria in the total microbial population. Other found organisms included *Anellida*, *Nematoda*, and *Platyhelminthes* metazoans, and DNA viruses (*Cossaviricota*, *Uroviricota*, *Cressdnaviricota*, *Nucleocytoviricota*; alltogether representing the 0.01% of the total detected phyla). Unfortunately, due to the extraction technique, sand samples were not available for the search of RNA viruses, which instead could be present and should be analyzed in more detail, based on their reported presence in beaches [35].

Interestingly, the resistome analysis of the sand microbiome evidenced the presence of antibiotic resistance determinants conferring the resistance against aminoglycoside (*AA1* group), macrolides, streptogramin, and lincosamides (*ermA*, *ermB*, *mefA*), frequently detected in *Enterococcus* and *Staphylococcus* genera, vancomycin (*vanC*), mostly prevalent in Enterococci and particularly in *E. gallinarum*, confirming the diffusion and detectability of significant amount of resistance-genes in bacteria detected in beach sand [36,37,38].

Based on the significant presence of human bacterial, fungal, and viral potential pathogens in the analyzed beach sand, we assessed the potential of quicklime as a possible remediation tool. More specifically, the effect of the addition of 1% to 3% (*w*/*w*) of CaO to the sand was investigated, both in vitro and on field. The results showed that the addition of small amounts of CaO induced an instantaneous and remarkable decrease of the microbial population hosted in the sand. In detail, CaO inactivated >99% of bacteria and fungi within 1 day in vitro, at all tested concentrations. Due to the reported presence of viruses in sand [35,39], also detected by us, the CaO actin was also assessed against high loads of the resistant MVA and EV71 viruses, showing their complete inactivation within 1 h of contact with CaO, in in vitro assays performed on artificially contaminated sand.

The addition of CaO to sand samples in the presence of 15% of water induced immediate heating and pH raise toward alkalinity, likely responsible for microbes’ inactivation. Notably, CaO usage was also recently tested and proposed for food decontamination [40].

When moving to on-field tests, in controlled or supervised conditions, more like what could be observed in real-life conditions, CaO action maintained its effectiveness, decreasing the microbial aerobic and anaerobic population up to 90% in a concentration-dependent manner, and maintaining stably low the total CFU number till 35 days in controlled conditions and 26 days in open field, where the recontamination phenomena were allowed. Interestingly, the profile of sand microbiome did not appear significantly altered at a qualitative level, as most phyla and genera were present in both untreated controls and CaO-treated sand. Rather, all the microbial components present in control sand were quantitatively reduced by CaO, without substantial modifications of their relative abundance. However, some significant changes were observed in the relative abundance of some core species, including *Cutibacterium acnes*, *Nocardioides* spp., *Priestia flexa*, *Sphingomonas koreensis*, *Pseudarthrobacter phenanthrenivorans*, *Pseudomonas aeruginosa*/*stutzeri*, *Bordetella petrii*, and *Microbacterium* sp. *Y-01*. Further species were decreased or virtually absent following CaO treatment (*Ensifer adherens*, *Achromobacter spanius*, *Tardibacter chloroacetimidivorans*, *Chroococcidioides thermalis*, *Streptomyces rubulavendulae*, *Metabacillus litoralis*, *Ralstonia pickettii*, *Arthrobacter YN*), although the differences were not statistically significant compared to controls, due to the naturally low relative abundance of these core species in controls. By contrast, *Priestia megaterium* and *Streptomyces alfalfae* resulted increased in CaO-treated vs. untreated samples.

Of note, both increased core bacteria are not correlated to infection in humans, being environmental bacteria mostly promoting plant growth [41]. Some of the reduced core species are instead associated with human diseases, including *Nocardioides* spp., *Pseudomonas* spp., *Cutibacterium* spp., *Staphylococcus* spp., and *Micrococcus* spp. Besides the already mentioned opportunistic pathogens, *Nocardioides* can sustain infections affecting lungs, brain, and skin [42]. Such bacteria can be transmitted by breathing dust containing bacteria and by skin contact through a cut or scrape, causing a potentially disseminated infection which can be lethal in immunosuppressed [42].

Limitations of the study include the period during which the analyses were performed (November–June), since the microbial contamination of human origin is expected to increase significantly during the summer season, due to the increased presence of people on the beaches [43]. Thus, the study should be extended to summer period to get generalizable results.

This study highlights the importance of holistic management of beaches, putting evidence on beach sand monitoring besides water quality monitoring, and provides the first evidences on the possible use of CaO for sand decontamination purposes. We are aware that quicklime needs wise management decisions before using either in the terrestrial or aquatic environment, since in water at high concentrations can be dangerous to mollusks and fish [44]. Compared to the CaO concentrations used to treat sludge from wastewater treatment plants (up to 20%, *w*/*v*) [14], the amounts used in this study are much lower. Nevertheless, precautions are needed before applying it specifically as a disinfectant, and more studies are needed on its potential application in beach sand. However, treating the sand in the backside of the coast would not put active CaO in contact with water and benthic alive organisms, preserving the life of mollusks and fishes; moreover, CaO effects results totally reversible in around 40 days, and no effect was observed associated with the exhaust product, calcium carbonate. These data, providing further studies on any eventual induced modification in the sea water close to the coast, may open the way to consider CaO as an effective, low-cost, and eco-sustainable way to restore the microbiological quality of sand in highly contaminated areas.

## 5. Conclusions

While coastal waters have long been monitored for the presence of potential human pathogens to evaluate the infectious risk for people frequenting the beaches, the sand microbiome has received much less attention. Our study confirms previous data collected by others in the recent years [45,46], demonstrating the presence of several potential human pathogens in the beach sand. This highlights the importance of monitoring also beach sand beside water, to define the biological quality and safety of beaches [47]. Collected data, obtained for the first time by simultaneous culture-based and molecular analyses, showed the presence of bacteria of likely fecal origin (*Enterobacteriaceae* and *Enterococci*) at a very high level compared to what is considered the threshold of attention in marine water and in beach sand [29]. Most of them were probably of animal origin, highlighting the potential pollutant impact of animal farms on the beach sand natural flora. Of note, significant amount of resistance-genes were detected in bacteria inhabiting the beach sand, confirming their spread in any environment.

Our data also provide first evidence of the effectiveness of very low concentrations of quicklime for sand decontamination, suggesting its possible usage to restore the microbiological safety in highly contaminated sands, where the risk of transmission of potentially harmful pathogens is high. However, since it is obviously important to maintain the balance of the natural beach ecosystem, future studies addressed to evaluate any eventual impact of quicklime on the benthic coastal fauna/flora are needed. By providing such data, quicklime may represent a valuable and sustainable tool to restore a good microbiological quality in highly contaminated beach areas.

## Figures and Tables

**Table 1:** CFU count of the cultivable microbes searched in sand.

Genus/Species	CFU/g ^1^
*Pseudomonas* spp.	1.6 × 10^5^ ± 1.1 × 10^3^
*Staphylococcus* spp.	1.9 × 10^4^ ± 6.7 × 10^2^
*Enterococcus* spp.	1.1 × 10^3^ ± 1.9 × 10^2^
*Enterobacteriaceae* spp.	4.4 × 10^3^ ± 2.2 × 10^2^
*Clostridium* spp.	1.5 × 10^4^ ± 1.3 × 10^2^
*Fungi*	4.8 × 10^2^ ± 5.4 × 10

^1^ The results are expressed as the mean CFU ± S.D. number per gram of sand, obtained in duplicate samples of two consecutive serial dilutions.

**Table 2:** Microbial species detected by qPCR microarray in beach sand samples.

Species	Positivitity	Ct ^1^
*Arcobacter butzleri*	++	28.94
*Arcobacter skirrowii*	+	31.00
*Bifidobacterium adolescentis*	+	30.04
*Campylobacter upsaliensis*	+	30.97
*Clostridium perfringens*	+/−	36.82
*Desulfovibrio desulfuricans*	++	29.42
*Enterococcus gallinarum*, *Enterococcus casseliflavus*	++	26.91
*Enterococcus faecalis*	+/−	37.50
*Enterococcus faecium*	+/−	37.16
*Morganella morganii*	++	29.87
*Ruminococcus bromii*	+	33.27
*Shigella dysenteriae* (*Salmonella bongori*)	++	27.83
*Streptococcus suis*	+/−	36.98
*Vibrio choleare*	++	22.74
*Vibrio vulnificus*	+	34.07
*Yersinia enterocolitica*	+	31.41
*eae* (*intimin adherence protein*)	++	29.09
*stx2A* (*Shiga-like toxin II*)	+/−	37.99

^1^ Results are expressed as the average Ct (threshold cycles) values obtained in duplicate samples processed by a 40-cycles amplification reaction. +/−, Ct >35; +, 30 ≤ Ct ≤ 35; ++, Ct < 30.

**Table 3:** pH values in CaO-treated sand samples ^1^.

Condition	1 h	2 h	1 d	2 d
CTR (untreated)	9.38	9.01	8.84	9.28
CaO 1%	12.40	12.31	11.38	9.57
CaO 2.5%	12.23	12.80	11.97	10.85
CaO 5%	12.68	12.72	12.07	12.02

^1^ The supernatant of control and treated sand samples was assessed as described in Methods at the indicated times post-contact with CaO at the indicated concentrations.

**Table 4:** pH values in CaO-treated sand: on field assays ^1^.

Condition	1 h	2 h	1 d	2 d	7 d	14 d	26 d	47 d	63 d	100 d
CTR	9.04	8.98	9.01	9.08	9.73	8.99	9.01	8.96	9.05	9.07
CaO 1%	**11.34**	**11.45**	**11.98**	**12.01**	**12.22**	**11.01**	8.89	8.85	9.05	8.89
CaO 2%	**12.04**	**12.21**	**12.30**	**12.38**	**12.36**	**11.54**	8.87	8.95	9.03	8.99
CaO 2% (n.a.w.)	**12.26**	**12.31**	**12.32**	**12.25**	**12.36**	**11.31**	9.05	8.86	8.99	9.02
CaO 3%	**12.09**	**12.42**	**12.45**	**12.40**	**12.43**	**11.67**	**11.21**	9.67	9.06	9.01
CaCO_3_ 3%	8.99	9.01	9.10	8.98	8.96	8.96	8.95	8.74	8.95	8.92

^1^ The supernatant of untreated control and CaO-treated sand samples was assessed as described in Methods at the indicated timepoints, from 1 h to 100 days after contact. n.a.w., no added water. Significantly increased pH values, compared to controls, are indicated in bold (*p* < 0.05).

**Table 5:** Core species detected in control and CaO-treated beach sand.

Core Species	Mean RA (%) ^1^	
	CTR	CaO ^2^
*Priesta megaterium*	11.53	13.97
*Streptomyces alfalfae*	6.36	8.01
*Pseudomonas putida*	3.57	3.25
*Cutibacterium acnes*	3.06	0.44
*Nocardioides* sp. *CF8*	4.31	0.76
*Priestia flexa*	3.46	0.94
*Fusarium oxysporum*	0.32	0.35
*Nocardioides dokdonensis*	3.80	0.46
*Sphingomonas koreensis*	3.13	0.36
*Pseudarthrobacter phenanthrenivorans*	2.07	0.24
*Bacillus halotolerans*	1.33	0.51
*Pseudomonas aeruginosa*	0.34	-
*Sinorhizobium meliloti*	0.79	0.94
*Pseudomonas stutzeri*	1.26	0.24
*Bacillus circulans*	0.90	0.09
*Bordetella petrii*	1.20	-
*Ensifer adhaerens*	0.17	-
*Achromobacter spanius*	0.65	-
*Rhodococcus hoagii*	0.59	0.55
*Sphingopyxis macrogoltabida*	0.51	0.71
*Arthrobacter* sp. *ZXY-2*	0.65	-
*Microbacterium* sp. *Y-01*	1.95	-
*Nocardioides euryhalodurans*	0.19	-
*Cellulosimicrobium* sp. *TH-20*	1.06	-
*Tardibacter chloracetimidivorans*	1.11	-
*Nocardioides seonyuensis*	0.11	-
*Chroococcidiopsis thermalis*	0.008	-
*Pseudorhizobium banfieldiae*	0.60	0.37
*Nocardioides* sp. *JS614*	0.84	-
*Priestia filamentosa*	0.23	0.15
*Achromobacter* sp. *MFA1 R4*	0.47	0.50
*Streptomyces rubrolavendulae*	0.62	-
*Metabacillus litoralis*	0.30	-
*Ralstonia pickettii*	0.37	-
*Arthrobacter* sp. *YN*	0.21	-
*Tsuneonella amylolytica*	0.05	0.11
*Paenibacillus mucilaginosus*	0.15	0.03

^1^ Results are expressed as the average percentage of relative abundance (RA) obtained for each indicated species at all times tested. ^2^ CaO results refer to the samples treated with CaO 3% (*w*/*w*).

**Table 6:** Alpha-diversity index in control and CaO-treated beach samples.

Time	Sample	Alpha-Index ^1^	CaO/CTR Alpha-Index ^2^	*p*
1 h	CTR	8.86	1.00	n.s.
	CaO 1%	7.89	0.89	n.s.
	CaO 2%	8.41	0.95	n.s.
	CaO 3%	8.90	1.00	n.s.
1 d	CTR	10.52	1.00	n.s.
	CaO 1%	9.96	0.94	n.s.
	CaO 2%	8.56	0.81	n.s.
	CaO 3%	8.01	**0.76**	0.05
2 d	CTR	13.61	1.00	n.s.
	CaO 1%	12.59	0.92	n.s.
	CaO 2%	11.33	0.83	n.s.
	CaO 3%	10.69	**0.78**	0.05
7 d	CTR	10.09	1.00	n.s.
	CaO 1%	8.62	0.85	n.s.
	CaO 2%	7.79	**0.77**	0.05
	CaO 3%	5.57	**0.55**	0.01
14 d	CTR	13.72	1.00	n.s.
	CaO 1%	12.75	0.92	n.s.
	CaO 2%	10.01	**0.72**	0.05
	CaO 3%	6.01	**0.44**	0.01
35 d	CTR	11.49	1.00	n.s.
	CaO 1%	11.14	0.96	n.s.
	CaO 2%	10.92	0.95	n.s.
	CaO 3%	9.67	0.84	n.s.
63 d	CTR	12.24	1.00	n.s.
	CaO 1%	11.91	0.97	n.s.
	CaO 2%	12.24	1.00	n.s.
	CaO 3%	12.82	1.04	n.s.

^1^ Results are expressed as alpha-index values calculated based on the WGS data obtained in the indicated samples. ^2^ CaO/CTR alpha-index values represent the ratio between CaO-treated and control sample values, which were taken as 1.00.

## Data Availability

Raw sequencing data and bioinformatics analyses have been deposited in the European Nucleotide Archive (ENA) website (accession number PRJEB61323).

## References

[1] Valerio E., Santos M.L., Teixeira P., Matias R., Mendonca J., Ahmed W., Brandao J. (2022). Microbial source tracking as a method of determination of beach sand contamination. Int. J. Environ. Res. Public Health.

[2] Tamponi C., Knoll S., Tosciri G., Salis F., Dessi G., Cappai M.G., Varcasia A., Scala A. (2020). Environmental contamination by dog feces in touristic areas of Italy: Parasitological aspects and zoonotic hazards. Am. J. Trop. Med. Hyg..

[3] Ristic M., Miladinovic-Tasic N., Dimitrijevic S., Nenadovic K., Bogunovic D., Stepanovic P., Ilic T. (2020). Soil and sand contamination with canine intestinal parasite eggs as a risk factor for human health in public parks in Nis (Serbia). Helminthologia.

[4] Sidhu J.P., Ahmed W., Gernjak W., Aryal R., McCarthy D., Palmer A., Kolotelo P., Toze S. (2013). Sewage pollution in urban stormwater runoff as evident from the widespread presence of multiple microbial and chemical source tracking markers. Sci. Total Environ..

[5] Sidhu J.P., Hodgers L., Ahmed W., Chong M.N., Toze S. (2012). Prevalence of human pathogens and indicators in stormwater runoff in Brisbane, Australia. Water Res..

[6] Nayak B., Weidhaas J., Harwood V.J. (2015). La35 poultry fecal marker persistence is correlated with that of indicators and pathogens in environmental waters. Appl. Environ. Microbiol..

[7] Zeki S., Aslan A., Burak S., Rose J.B. (2021). Occurrence of a human-associated microbial source tracking marker and its relationship with faecal indicator bacteria in an urban estuary. Lett. Appl. Microbiol..

[8] McQuaig S., Griffith J., Harwood V.J. (2012). Association of fecal indicator bacteria with human viruses and microbial source tracking markers at coastal beaches impacted by nonpoint source pollution. Appl. Environ. Microbiol..

[9] Halliday E., McLellan S.L., Amaral-Zettler L.A., Sogin M.L., Gast R.J. (2014). Comparison of bacterial communities in sands and water at beaches with bacterial water quality violations. PLoS ONE.

[10] Akanbi O.E., Njom H.A., Fri J., Otigbu A.C., Clarke A.M. (2017). Antimicrobial susceptibility of *Staphylococcus aureus* isolated from recreational waters and beach sand in eastern cape province of South Africa. Int. J. Environ. Res. Public Health.

[11] Maciel N.O., Johann S., Brandao L.R., Kucharikova S., Morais C.G., Oliveira A.P., Freitas G.J., Borelli B.M., Pellizzari F.M., Santos D.A. (2019). Occurrence, antifungal susceptibility, and virulence factors of opportunistic yeasts isolated from brazilian beaches. Mem. Inst. Oswaldo. Cruz..

[12] Gerken T.J., Roberts M.C., Dykema P., Melly G., Lucas D., De Los Santos V., Gonzalez J., Butaye P., Wiegner T.N. (2021). Environmental surveillance and characterization of antibiotic resistant *Staphylococcus aureus* at coastal beaches and rivers on the island of Hawai’i. Antibiotics.

[13] Alm E.W., Zimbler D., Callahan E., Plomaritis E. (2014). Patterns and persistence of antibiotic resistance in faecal indicator bacteria from freshwater recreational beaches. J. Appl. Microbiol..

[14] Malcheva B.Z., Petrov P.G., Stefanova V.V. (2022). Microbiological control in decontamination of sludge from wastewater treatment plant. Processes.

[15] Caselli E., Brusaferro S., Coccagna M., Arnoldo L., Berloco F., Antonioli P., Tarricone R., Pelissero G., Nola S., La Fauci V. (2018). Reducing healthcare-associated infections incidence by a probiotic-based sanitation system: A multicentre, prospective, intervention study. PLoS ONE.

[16] D’Accolti M., Soffritti I., Bini F., Mazziga E., Cason C., Comar M., Volta A., Bisi M., Fumagalli D., Mazzacane S. (2023). Shaping the subway microbiome through probiotic-based sanitation during the COVID-19 emergency: A pre-post case-control study. Microbiome.

[17] Caselli E., Arnoldo L., Rognoni C., D’Accolti M., Soffritti I., Lanzoni L., Bisi M., Volta A., Tarricone R., Brusaferro S. (2019). Impact of a probiotic-based hospital sanitation on antimicrobial resistance and hai-associated antimicrobial consumption and costs: A multicenter study. Infect. Drug. Resist..

[18] Soffritti I., D’Accolti M., Fabbri C., Passaro A., Manfredini R., Zuliani G., Libanore M., Franchi M., Contini C., Caselli E. (2021). Oral microbiome dysbiosis is associated with symptoms severity and local immune/inflammatory response in COVID-19 patients: A cross-sectional study. Front. Microbiol..

[19] Caselli E., D’Accolti M., Vandini A., Lanzoni L., Camerada M.T., Coccagna M., Branchini A., Antonioli P., Balboni P.G., Di Luca D. (2016). Impact of a probiotic-based cleaning intervention on the microbiota ecosystem of the hospital surfaces: Focus on the resistome remodulation. PLoS ONE.

[20] D’Accolti M., Soffritti I., Mazzacane S., Caselli E. (2019). Fighting amr in the healthcare environment: Microbiome-based sanitation approaches and monitoring tools. Int. J. Mol. Sci..

[21] Caselli E., D’Accolti M., Soffritti I., Lanzoni L., Bisi M., Volta A., Berloco F., Mazzacane S. (2019). An innovative strategy for the effective reduction of mdr pathogens from the nosocomial environment. Adv. Exp. Med. Biol..

[22] Soffritti I., D’Accolti M., Cason C., Lanzoni L., Bisi M., Volta A., Campisciano G., Mazzacane S., Bini F., Mazziga E. (2022). Introduction of probiotic-based sanitation in the emergency ward of a children’s hospital during the COVID-19 pandemic. Infect. Drug. Resist..

[23] D’Accolti M., Soffritti I., Bonfante F., Ricciardi W., Mazzacane S., Caselli E. (2021). Potential of an eco-sustainable probiotic-cleaning formulation in reducing infectivity of enveloped viruses. Viruses.

[24] Donnenberg M.S., Tzipori S., McKee M.L., O’Brien A.D., Alroy J., Kaper J.B. (1993). The role of the eae gene of enterohemorrhagic *Escherichia coli* in intimate attachment in vitro and in a porcine model. J. Clin. Investig..

[25] Makino K., Yokoyama K., Kubota Y., Yutsudo C.H., Kimura S., Kurokawa K., Ishii K., Hattori M., Tatsuno I., Abe H. (1999). Complete nucleotide sequence of the prophage vt2-sakai carrying the verotoxin 2 genes of the enterohemorrhagic *Escherichia coli* o157:H7 derived from the sakai outbreak. Genes Genet Syst..

[26] Whitman R., Harwood V.J., Edge T.A., Nevers M., Byappanahalli M., Vijayavel K., Brandao J., Sadowsky M.J., Alm E.W., Crowe A. (2014). Microbes in beach sands: Integrating environment, ecology and public health. Rev. Environ. Sci. Biotechnol..

[27] Buczek M., ASM (2017). Sandy Beach Microbes: The Good, the Bad, and the Flesh-Eating.

[28] Pianetti A., Bruscolini F., Sabatini L., Colantoni P. (2004). Microbial characteristics of marine sediments in bathing area along Pesaro-Gabicce Coast (Italy): A preliminary study. J. Appl. Microbiol..

[29] WHO (2021). Recreational Water Quality Guidelines. https://www.who.int/news/item/13-07-2021-who-launches-guidelines-for-recreational-water-quality-as-summer-heats-up.

[30] Turnbull J.D., Russell J.E., Fazal M.A., Grayson N.E., Deheer-Graham A., Oliver K., Holroyd N., Parkhill J., Alexander S. (2019). Whole-genome sequences of five strains of *Kocuria rosea*, nctc2676, nctc7514, nctc7512, nctc7528, and nctc7511. Microbiol. Resour. Announc..

[31] Elston M.J., Dupaix J.P., Opanova M.I., Atkinson R.E. (2019). *Cutibacterium acnes* (formerly *Proprionibacterium acnes*) and shoulder surgery. Hawaii J. Health Soc. Welf..

[32] Zhu M., Zhu Q., Yang Z., Liang Z. (2021). Clinical characteristics of patients with micrococcus luteus bloodstream infection in a chinese tertiary-care hospital. Pol. J. Microbiol..

[33] Severn M.M., Horswill A.R. (2023). *Staphylococcus epidermidis* and its dual lifestyle in skin health and infection. Nat. Rev. Microbiol..

[34] Mueller M., Tainter C.R. (2023). Escherichia coli.

[35] Solo-Gabriele H., Harwood V., Kay D., Fujioka R., Sadowsky M., Whitman R., Brandão J. (2016). Beach sand and the potential for infectious disease transmission: Observations and recommendations. J. Mar. Biol. Assoc. UK.

[36] Andrade Vda C., Zampieri Bdel B., Ballesteros E.R., Pinto A.B., de Oliveira A.J. (2015). Densities and antimicrobial resistance of *Escherichia coli* isolated from marine waters and beach sands. Environ. Monit. Assess..

[37] Fernandes Cardoso de Oliveira A.J., Ranzani de Franca P.T., Pinto A.B. (2010). Antimicrobial resistance of heterotrophic marine bacteria isolated from seawater and sands of recreational beaches with different organic pollution levels in southeastern Brazil: Evidences of resistance dissemination. Environ. Monit. Assess..

[38] de Oliveira A.J., Pinhata J.M. (2008). Antimicrobial resistance and species composition of *Enterococcus* spp. Isolated from waters and sands of marine recreational beaches in southeastern Brazil. Water Res..

[39] Carducci A., Federigi I., Balestri E., Lardicci C., Castelli A., Maltagliati F., Zhao H., Menicagli V., Valente R., De Battisti D. (2022). Virus contamination and infectivity in beach environment: Focus on sand and stranded material. Mar. Pollut Bull..

[40] Choi H.Y., Bang I.H., Kang J.H., Min S.C. (2019). Development of a microbial decontamination system combining washing with highly activated calcium oxide solution and antimicrobial coating for improvement of mandarin storability. J. Food Sci..

[41] Chen J., Hu L., Chen N., Jia R., Ma Q., Wang Y. (2021). The biocontrol and plant growth-promoting properties of streptomyces alfalfae xn-04 revealed by functional and genomic analysis. Front. Microbiol..

[42] CDC (2016). Nocardiosis. https://www.cdc.gov/nocardiosis/index.html.

[43] Toubiana M., Salles C., Tournoud M.G., Licznar-Fajardo P., Zorgniotti I., Tremelo M.L., Jumas-Bilak E., Robert S., Monfort P. (2021). Monitoring urban beach quality on a summer day: Determination of the origin of fecal indicator bacteria and antimicrobial resistance at Prophete Beach, Marseille (France). Front. Microbiol..

[44] El-Mansy A. (2020). Evaluation of the effect of quicklime on some organisms from different ecosystems in Egypt: Morphological perspective. World Environ..

[45] De Giglio O., Narracci M., Apollonio F., Triggiano F., Acquaviva M.I., Caroppo C., Diella G., Di Leo A., Fasano F., Giandomenico S. (2022). Microbiological and chemical characteristics of beaches along the Taranto Gulf (Ionian Sea, southern Italy). Environ. Monit. Assess..

[46] Bonanno Ferraro G., Suffredini E., Mancini P., Veneri C., Iaconelli M., Bonadonna L., Montagna M.T., De Giglio O., La Rosa G. (2021). Pepper mild mottle virus as indicator of pollution: Assessment of prevalence and concentration in different water environments in Italy. Food Environ. Virol..

[47] Brandao J., Valerio E., Weiskerger C., Verissimo C., Sarioglou K., Novak Babic M., Solo-Gabriele H.M., Sabino R., Rebelo M.T. (2023). Strategies for monitoring microbial life in beach sand for protection of public health. Int. J. Environ. Res. Public Health.

